# *Rambellisea gigliensis* and *Rambellisea halocynthiae*, gen. et spp. nov. (Lulworthiaceae) from the Marine Tunicate *Halocynthia papillosa*

**DOI:** 10.3390/jof10020127

**Published:** 2024-02-03

**Authors:** Martina Braconcini, Susanna Gorrasi, Massimiliano Fenice, Paolo Barghini, Marcella Pasqualetti

**Affiliations:** 1 Department of Ecological and Biological Sciences (DEB), University of Tuscia, 01100 Viterbo, Italy; martina.braconcini@unitus.it (M.B.); gorrasi@unitus.it (S.G.); fenice@unitus.it (M.F.); barghini@unitus.it (P.B.); 2 Laboratory of Applied Marine Microbiology, CoNISMa, Department of Ecological and Biological Sciences, University of Tuscia, 01100 Viterbo, Italy; 3 Laboratory of Ecology of Marine Fungi, CoNISMa, Department of Ecological and Biological Sciences, University of Tuscia, 01100 Viterbo, Italy

**Keywords:** marine fungi, new taxa, *Rambellisea*, epizoic fungi, *Halocynthia papillosa*

## Abstract

In this study, 15 Lulworthiales strains isolated from the marine tunicate *Halocynthia papillosa* collected in the central Tyrrhenian Sea were characterized using a polyphasic approach (morpho-physiological, molecular, and phylogenetic analyses). Based on multi-locus phylogenetic inference and morphological characters, a new genus, *Rambellisea*, and two new species, *R. halocynthiae* and *R. gigliensis* (Lulworthiales), were proposed. Multi-locus phylogenetic analyses using the nuclear ribosomal regions of DNA (nrITS1-nr5.8S-nrITS2, nrLSU, and nrSSU) sequence data strongly supported the new taxa. Phylogenetic inference, estimated using Maximum Likelihood and Bayesian Inference, clearly indicates that *Rambellisea* gen. nov. forms a distinct clade within the order Lulworthiales. Moreover, the two new species were separated into distinct subclades, solidly supported by the analyses. This is the first report of Lulworthiales species isolated from animals.

## 1. Introduction

Marine habitats cover more than 70% of the planet’s surface and host a large amount of unknown biological and chemical diversity [1,2,3,4]. The sea represents a limitless resource of unexploited substrata and new microorganisms [5,6,7,8]. Over the last decades, researchers have paid close attention to marine microbiology, investigating new environments and/or substrata [6,9,10]. In particular, scientists have spent much effort to expand our understanding of fungal biodiversity and highlight fungi’s importance in several ecosystem services [6,11,12,13,14].

The total number of fungi from marine environments, reported on the “Marine Fungi” specialized website [15], accounts for 1947 species (30 October 2023). However, it has been estimated that this is only a small fraction (<0.2%) of the total marine fungal diversity [11]. To date, up to 90% of marine described species belong to the phyla Ascomycota and Basidiomycota [15]. With more than 940 species and 385 genera, Ascomycota is the most common taxon, and the greatest number of species are found in the classes Dothideomycetes, Eurotiomycetes, and Sordariomycetes [16]. Some orders of Sordariomycetes are exclusively or preferentially marine: Koralionastetales and Lulworthiales host only marine species [17], whereas the Halosphaeriaceae family includes both freshwater and marine species, even though the marine ones are the most numerous [18,19].

The order Lulworthiales, with the single family Lulworthiaceae, was established by Kohlmeyer et al. [20] based on phylogenetic analyses and morphological characters to accommodate the genera *Lulworthia* and *Lindra*, previously included in the order Halosphaeriales. Over the past few years, several new marine fungi have been described in the family (*Lulworthia atlantica,* and *L. fundyensis)*, including the recently established genus *Paralulworthia*, in 2020. This genus was established to accommodate five new species that were discovered in *Posidonia oceanica* (*P. gigaspora, P. posidoniae, P. candida, P. elbensis*, and *P. mediterranea*) [21,22,23,24,25]. The species *P. candida, P. elbensis*, and *P. mediterranea*, which do not produce reproductive structures, were established solely based on phylogenetic analyses [23]. It should be noted that several marine fungal strains exhibit only *mycelia sterilia,* and they could be identified exclusively by molecular approach [5,26,27]. This approach, although significantly different from the traditional taxonomy based on reproductive characterization, is now widely accepted by the scientific community [28]. In the last few years, several new species and genera were established in the absence of sexual or asexual structures [29,30,31,32].

The Lulworthiaceae family is characterized by filamentous ascospores [20]; nevertheless, for some recently described species, reproductive structures have not been observed. The family comprises 80 species and 16 genera (https://www.indexfungorum.org/, accessed on 17 September 2023; https://www.mycobank.org/, accessed on 17 September 2023) [33]. Members of the family have a cosmopolitan distribution and live in a wide range of habitats, including drifts, submerged woods, algae, and seagrasses [20,24,34,35,36,37,38,39,40,41]. Some species have also been reported from polluted water, such as those from oil-spilled areas [42]. To the best of our knowledge, members of the order Lulworthiales have not yet been isolated from marine animals, despite some metabarcoding studies revealing the presence of some genera in association with various coral species. The species *L. calcicola* has been described from coral rock [43,44,45,46].

During a survey carried out in the central Tyrrhenian Sea to study epizoic fungi, some new strains belonging to Lulworthiales were isolated from the tunicate *Halocynthia papillosa*. *Halocynthia papillosa* is a common ascidian species inhabiting the Mediterranean Sea [47,48], and it presents a tunic composed of cellulose, acid mucopolysaccharides, proteins, and sulfated glycans [49]. Some of these compounds, such as cellulose, are extremely rare biomolecules in animals [50].

In this study, 15 Lulworthiales strains isolated from *H. papillosa* were characterized using a polyphasic approach (morpho-physiological, molecular, and phylogenetic analyses). Based on multi-locus phylogenetic inference and morphological characters, a new genus, *Rambellisea,* and two new species, *Rambellisea halocynthiae* and *Rambellisea gigliensis*, are here proposed.

## 2. Materials and Methods

### 2.1. Fungal Isolation

Five specimens of *H. papillosa* were collected near the “Punta Gabbianara” cape (42°21′50″ N–10°55′24″ E), Giglio Island (Tuscan Archipelago, North Tyrrhenian Sea) at 23–28 m depth in March 2022. The samples were placed in sterile containers and maintained at 4 °C. Isolations were carried out within 24 h as follows: Samples were washed in sterilized artificial seawater (SW; Sea Salts, 35 g dissolved in 1 L, Sigma-Aldrich, St. Louis, MO, USA) to eliminate debris and any potential transient propagules. For each animal, the tunic (T) was separated from the inner tissues (I) to evaluate mycobiota differences related to animal districts. For fungal isolation, the following two different techniques were used:

(i) Direct plating: tunic was cut into pieces of about 1 cm^3^ and directly plated (5 pieces for each plate) onto Petri dishes (90 mm) containing Malt Extract Agar seawater (MEAsw; 50 g MEA—Sigma-Aldrich dissolved in 1 L of seawater) and Corn Meal Agar seawater (CMAsw; 17 g CMA–Fluka analytical, Buchs, Switzerland, dissolved in 1 L of seawater).
(ii) Homogenization: 5 g of each district (T, I) was homogenized in 10 mL of sterile seawater using a sterile device (ULTRA-TURRAX, IKA, Staufen, Germany). A total of 500 μL of each suspension was plated onto Petri dishes (90 mm) containing MEAsw and CMAsw.

To avoid bacterial growth, all media were supplemented with antibiotics (Streptomycin Sulfate, 0.2 g/L; Penicillin G 0.07 g/L; Chloramphenicol, 0.05 g/L). All plates were incubated at 25 °C in the dark and checked daily for four weeks. Strains were isolated in axenic culture on CMAsw and cryogenically maintained at −40 °C in the culture collection of microorganisms of the “Laboratory of Ecology of Marine Fungi” (DEB, University of Tuscia, Viterbo, Italy). Samples of each species were also preserved at the Mycotheca Universitatis Taurinensis (MUT) culture collection.

The fungal strains analyzed in this study were HPa3, HPa15, HPa16, HPa50, HPa51, HPa52, HPa53, HPa54, HPa58, HPa59, HPa60, HPa61, HPa62, HPa63, and HPa64.

### 2.2. Morphology and Growth Studies on Different Media

Morphological analyses were carried out on plates utilizing different cultural media: Potato Dextrose Agar seawater (PDAsw; 39 g PDA—Sigma-Aldrich dissolved in 1 L of filtered seawater), Malt Extract Agar seawater (MEAsw), Corn Meal Agar seawater (CMAsw), and Oatmeal Agar seawater (OAsw; 30 g oatmeal powder, 20 g agar dissolved in 1 L of seawater).

The plates (5 cm or 9 cm Ø) were inoculated with a single agar disc (2 mm^2^) cut from the actively growing margin of 14 d strain cultures on PDAsw and incubated at 25 °C in sealed plastic boxes. These were humidified by a small beaker of distilled water to prevent evaporation and salt precipitation. Growth was monitored for 28 days, and the macroscopic and microscopic features were annotated.

To promote reproduction, fungal strains were inoculated on different natural substrata, such as bark (*Quercus cerris*), wood (*Pinus pinaster*), and tunic of *H. papillosa* (substrate of isolation). All substrata were sterilized, cut into small pieces (3 × 1 cm), and transferred to the surface of PDAsw well-developed colonies (21 days old). The plates were incubated for 4 weeks at 25 °C to allow natural substrata colonization. Following that, some of the inoculated fragments were transferred into tubes containing 20 mL of sterile seawater to simulate natural conditions, while others were transferred to moist chambers and further incubated for 4 months. All inoculated fragments were checked regularly.

The strains’ growth preference in relation to salinity was also investigated: each strain was inoculated, as mentioned above, on PDA plates (5 cm Ø) supplemented with different amounts of NaCl (0, 30, 50, 70, 80, and 100‰). The growth diameter was measured daily for 21 days. All experiments were carried out in triplicate.

### 2.3. DNA Extraction, PCR Amplification, and Data Assembling

Genomic DNA was extracted from fresh mycelium (about 100 mg) using the ZR Fungal/Bacterial DNA MiniPrep Kit (Zymo Research, Irvine, CA, USA), according to the manufacturer’s directions. The extracted DNA was spectrophotometrically quantified (Qubit, Thermo Fisher Scientific, Waltham, MA, USA) and stored at −20 °C.

For each fungal strain, the ITS1-5.8S-ITS2, LSU, and SSU of rDNA regions were amplified using the primer pairs ITS5/ITS4 [51], LR0R/LR7 [52], and NS1/NS4 [51], respectively. Amplifications were run in a 2720 Thermal Cycler (Applied Biosystem, Waltham, MA, USA) programmed as described in Table 1.

Polymerase chain reactions (PCR) were performed in a volume of 25 μL mixture containing 0.5 μL of each primer (10 μM), 2.5 μL of MgCl_2_ (25 mM), 1.5 μL of 5× buffer, 0.5 μL of dNTPs (10 mM), 0.2 μL of Go-Taq Polymerase (Promega, Madison, WI, USA), and 2 μL of genomic DNA; the final volume (25 μL) was reached by adding ultrapure water. The PCR products were purified (E.Z.N.A. Cycle Pure kit Omega Bio-tek, Norcross, GA, USA) and sent to Eurofins Genomics (Ebersberg, Germany) for sequencing. The sequences obtained were checked and trimmed with the Chromas Lite 2.1 program and then compared with those deposited in GenBank NCBI (National Center for Biotechnology Information, Bethesda, MD, USA). Newly generated sequences were deposited in GenBank (Table 2).

### 2.4. Sequence Alignment and Phylogenetic Analyses

For the phylogenetic analyses, a concatenated dataset of nrSSU, nrITS, and nrLSU sequences (Table 2) based on BLASTn results including the most representative species of the Lulworthiales genera according to the literature was used [21,22,23,24,25]. The single gene sequence datasets were aligned with the Clustal X 2.1 software [53] using the default parameters for gap opening and gap extension. Alignments were checked and edited using BioEdit Alignment Editor 7.2.5 [54] and manually adjusted in MEGA 10.2.6 when necessary. Positions where one or more species had a long mutation, as well as ambiguously aligned regions, were excluded from the subsequent phylogenetic analyses. The datasets were concatenated with MEGA X. Phylogenetic inference was estimated using Maximum Likelihood (ML) and Bayesian Inference (BI).

Maximum Likelihood analyses including 1000 bootstrap (BS) replicates were run using the IQ-TREE web server under different models for each dataset in the concatenated matrix [55]. ModelFinder on the IQ-TREE web server was used to determine the best nucleotide substitution model for each partition. TNe+G4 is the best-fit model for nrLSU, nr5.8S, and nrITS2, TIM2e+G4 for nrITS1, and TN+F+G4 for nrSSU [56]. The best scoring tree, with final likelihood values of −19719.747, was visualized using FigTree v.1.4 (http://tree.bio.ed.ac.uk/software/figtree/, accessed on 17 September 2023). The Bayesian Inference was performed with Mr Bayes 3.2.7 [57] under different models for each partition of the matrix as evaluated by jModelTest 2 [58] using Bayesian Information Criterion (TPM2+I+G for nrSSU part1 and TrN+G for nrSSUpart2; TIM1ef+G for nrITS1; TrNef+G for nr5.8S; TrN+G for nrITS2 and nrLSU). Substitution rates, gamma distribution shape parameter, and proportion of invariable sites were reported for each partition in Supplementary Materials (Table S1). The alignment was run for 1 million generations in two independent runs, each with four Markov Chains Monte Carlo (MCMC) and sampling every 100 iterations. As a “burn-in” measure, the first 25% of generated trees were discarded. MrBayes’ “sumt” function was used to generate a consensus tree, and Bayesian posterior probabilities (BYPP) were calculated.

Sequence alignment and phylogenetic tree were deposited in TreeBASE (www.treebase.org, accessed on 8 October 2023) (submission number: 30823). The new taxonomical names were recorded in Mycobank (MB850303, MB850305, MB850306).

## 3. Results

### 3.1. Phylogenetic Inference

A preliminary phylogenetic analysis was carried out individually for nrITS, nrLSU, and nrSSU. Since no incongruences were observed among the single-loci phylogenetic trees, a multi-locus analysis was performed thereafter. The dataset includes 84 strains, 29 species, and 11 genera belonging to the family Lulworthiaceae, with 3 pleosporelean species, *Bimuria novae-zelandiae*, *Setosphaeria monoceras*, and *Letendraea helminthicola,* as outgroup taxa. Globally, 26 sequences (15 nrITS, 5 nrSSU, and 6 nrLSU) were newly generated, whereas 186 were obtained from GenBank (Table 2).

The aligned concatenate dataset has 3346 characters, including gaps (1329 for SSU, 181 for ITS1, 150 for 5.8S, 310 for ITS2, and 1367 for LSU). Among them, 1598 distinct patterns, with 36.2% undetermined characters or gaps, 914 parsimony-informative sites, 434 singleton sites, and 1998 constant sites, were observed. Estimated base frequencies were A = 24.69%, T = 21.66%, C = 24.86%, and G = 28.80%.

**Figure 1:**
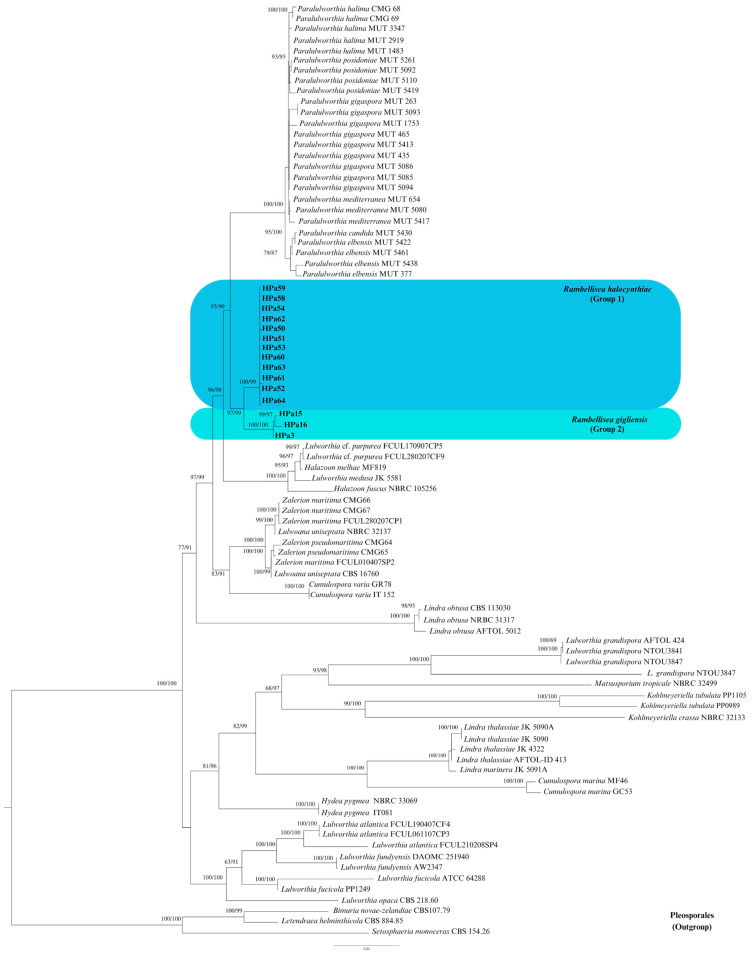
Phylogenetic inference based on combined nrITS, nrSSU, and nrLSU sequence dataset inferred using the Maximum Likelihood method. The tree is rooted to species of Pleosporales (*Bimuria novae-zelandiae*, *Setosphaeria monoceras*, and *Letendraea helminthicola*). Branch numbers indicate BS and BYPP values. Bar = expected changes per site (0.04). The strains resulting from the current study are in bold and the strains of each new species are distinguished by various colors.

ML analysis yielded a best-scoring three with a final optimization likelihood value of −19719.747. The ML and BI analyses resulted in generally congruent topologies, which were also in line with previous works [5,24]. Given the topological similarity of the two resulting trees, only the ML tree with BS and BYPP values was reported (Figure 1).

The 15 isolates under investigation formed a well-supported clade (BS = 97%; BYPP = 99%), constituting a new monophyletic lineage within the order Lulworthiales, with *Paralulworthia* species as their closest relatives (Figure 1). Within the new lineage, two groups can be distinguished, group 1: strains HPa50, HPa51, HPa52, HPa53, HPa54, HPa58, HPa59, HPa60, HPa61, HPa62, HPa63, and HPa64, and group 2: strains HPa3, HPa15, and HPa16. Both groups were strongly supported, with BS and BYPP values exceeding 99% (Group 1: BS = 100%; BYPP = 99%; Group 2: BS = 100%; BYPP = 100%).

The phylogenetic analysis appeared to support the conclusion that the fifteen strains isolated from *H. papillosa* belong to two novel species within a new genus in the Lulworthiaceae family (Figure 1).

The new genus *Rambellisea* is herein proposed, with the description of the following two new species: *Rambellisea halocynthiae* sp. nov. and *Rambellisea gigliensis* sp. nov.

Nucleotide divergence between the novel species and the closest was annotated for each locus when it occurred and reported as Supplementary Materials (Tables S2–S4).

### 3.2. Taxonomy

***Rambellisea*** Pasqualetti & Braconcini, **gen. nov.**

MycoBank no.: MB850303

**Etymology**. The prefix “*Rambelli*-”. In honor of the Italian Mycologist Angelo Rambelli, and the name “*Rambellisea*” refers to the genus habitat “sea”.

**Diagnosis**. Differs from the genus *Paralulworthia* to which it appears phylogenetically most closely related in the absence of sexual features and conidiogenous structures.

**Phylogenetic placement**. Lulworthiaceae, Lulworthiales, and Sordariomycetes. The genus *Rambellisea* gen. nov. clusters together with the genus *Paralulworthia* (Figure 1).

**Type species**. *Rambellisea gigliensis*.

**Figure 2:**
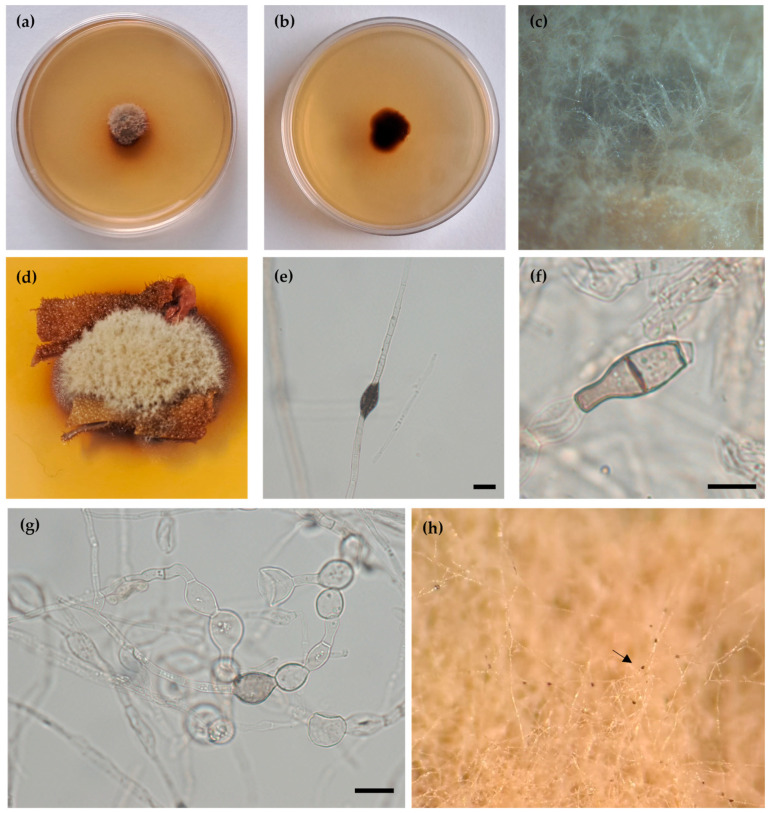
*Rambellisea gigliensis* sp. nov. HPa3 (MUT 6843) (**a**) 28-day-old colony on PDAsw (Ø 5 cm) at 25 °C (**b**) and reverse; (**c**) colony texture; (**d**) growth on *H. papillosa* tunic; (**e**) dark concretions on hyphae (MEAsw); (**f**,**g**) Chlamydospores; (**h**) exudates (arrow) produced on MEAsw. Scale bars: 10 μm (**e**,**f**), 20 μm (**g**).

***Rambellisea gigliensis*** Pasqualetti & Braconcini **sp. nov.** (Figure 2).

MycoBank no.: MB 850306.

**Etymology**. Referred to the sample collection site “Giglio Island”.

**Type**. Italy, Tuscany, Mediterranean Sea, Giglio Island (Grosseto), Punta Gabbianara, 42°21′50″ N, 10°55′24″ E, 25 m depth. Isolated from the tunic of *Halocynthia papillosa*, March 2022, Martina Braconcini. Holotype MUT 6843 (strain HPa3), living culture permanently preserved in a metabolically inactive state at MUT.

**Diagnosis**. *R. gigliensis* is an epizoic marine fungus. *R. gigliensis* (MUT 6843) differs from its closest phylogenetic neighbor *R. halocynthiae* (MUT 6851) by genetic characters in nrITS, nrLSU, and nSSU sequences (Tables S2–S4) and in the production of characteristically enlarged hyphae and chlamydospore production.

**Description.** Growing on *H. papillosa* tunic, *Q. cerris* bark, and *P. pinaster* wood.

Hyphae 3.0–4.6 μm wide, septate, sub-hyaline sometimes lightly pigmented, assuming a toruloid aspect mainly in submerged mycelium. In old cultures, dark concretions like small droplets were observed on hyphae (Figure 2e). Chlamydospores 10.5–20.0 μm, subhyaline to light brown, globose, sub-globose, monocellular, sometimes one septate, and pyriform (Figure 2f,g). Sexual and asexual structures not observed.

**Colony description**. Colonies on PDAsw, reaching 8 mm diameter after 28 days at 25 °C, dome-like, surface flocculose, smoke-grey to brown; aerial mycelium, whitish to light brown; margins regular, reverse brown. Soluble pigment is yellowish to orange, or absent exudates are absent (Figure 2a,b). Colonies on MEAsw, reaching 14 mm of diameter after 28 days at 25 °C, umbonate, surface floccose, beige to brown; aerial mycelium abundant, light brown; margins regular, reverse brown (Figure S1); soluble pigment absent; exudates present black in small droplets on aerial hyphae (Figure 2h). Colonies on CMAsw reached 32 mm in diameter after 28 days at 25 °C, plane slightly umbonate, surface velutinous, olive-grey to pale brown, margin regular submerged, aerial mycelium, whitish to light brown, mainly in the central area, reverse brown. Soluble pigment and exudates are not produced (Figure S1).

**Notes.** Based on a Megablast search on the NCBI nucleotide database, the closest hits of *R. gigliensis* (OR367423) using the nrITS are *R. halocynthiae* (GenBank accession no. OR36748; identities 492/541 (91%), 20 gaps), Lulworthiales sp. (GenBank accession no. LC544102; identities 468/549 (85%), 22 gaps), and *Zalerion* sp. (GenBank accession no. FJ430722; identities 411/468 (88%), 18 gaps). The closest hits using the nrLSU sequences are *R. halocynthiae* (GenBank accession no. OR371457; identities 1099/1115 (99%), 4 gaps), *P. posidoniae* (GenBank accession no. MZ357739; identities 1076/1107 (97%), 4 gaps), and *P. halima* (GenBank accession no. MZ357750; identities 1073/1103 (97%), 4 gaps). The closest hits using the nrSSU sequences are *R. halocynthiae* GenBank accession no. OR371485; identities 1039/1049 (99%), 0 gaps), *Lulworthia uniseptata* (GenBank accession no. AY879034; identities 1039/1050 (99%), 0 gaps), and *Z. maritima* (GenBank accession no. NG_078728; identities 1038/1050 (99%), 0 gaps). *R. gigliensis* isolates can be collected from the tunic and the internal tissues of *H. papillosa* and can be cultured on media with and without sea salt; the best growth was observed at the sea salinity on Corn Meal Agar (CMAsw).

**Additional material examined**. Italy, Tuscany, Mediterranean Sea, Giglio Island (Grosseto), Punta Gabbianara, 42°21′50″ N, 10°55′24″ E, 28 m depth. Isolated from the internal tissues of *H. papillosa*, March 2022, Martina Braconcini, living culture HPa15. Italy, Tuscany, Mediterranean Sea, Giglio Island (Grosseto), Punta Gabbianara, 42°21′50″ N, 10°55′24″ E, 23 m depth. Isolated from the internal tissues of *H. papillosa*, March 2022, Martina Braconcini, living culture HPa16.

**Figure 3:**
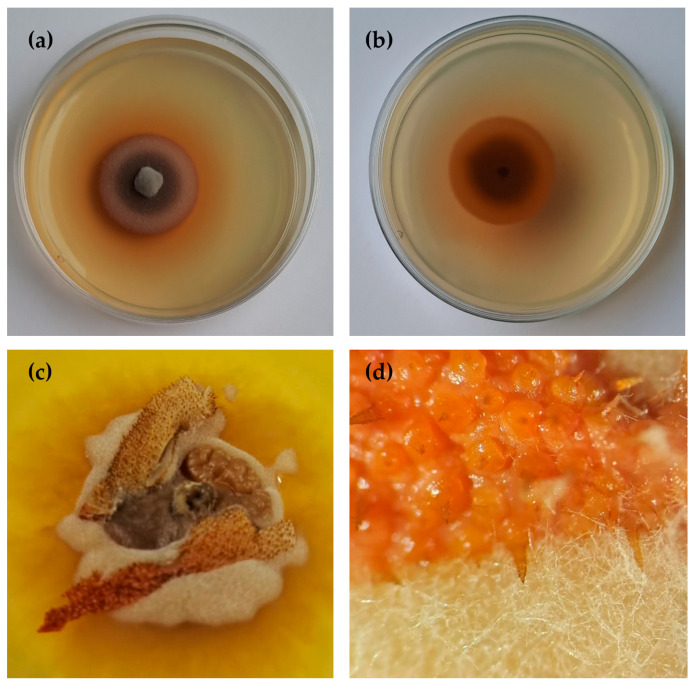
*Rambellisea halocynthiae* sp. Nov. Hpa52 (MUT 6851). (**a**) A 28-day-old colony on PDAsw (Ø 5 cm) at 25 °C (**b**) and reverse; (**c**,**d**) growth on *Halocynthia papillosa* tunic.

***Rambellisea halocynthiae*** Pasqualetti & Braconcini, **sp. nov**. (Figure 3).

MycoBank no.: MB850305.

**Etymology**. Referred to the substrate of isolation.

**Type**. Italy, Tuscany, Mediterranean Sea, Giglio Island (Grosseto), Punta Gabbianara, 42°21′50″ N, 10°55′24″ E, 25 m depth. Isolated from the tunic of *H. papillosa*, March 2022, Marcella Pasqualetti. Holotype MUT 6851 = HPa52, living culture permanently preserved in a metabolically inactive state at MUT.

**Diagnosis**. *R. halocynthiae* is an epizoic marine fungus. *R. halocynthiae* (MUT 6851) differs from its closest phylogenetic neighbor *R. gigliensis* (MUT 6843) by genetic characters in nrITS, nrLSU, and nSSU sequences (Tables S2–S4).

**Description**. Growing on *H. papillosa* tunic, *Q. cerris* bark, and *P. pinaster* wood.

Hyphae 2.2–4.4 μm wide, septate, sub-hyaline to slightly pigmented. Sexual and asexual structures are not observed.

**Colony description**. Colonies on PDAsw, reaching 13.5 mm in diameter after 28 days at 25 °C, plane centrally umbonate, surface velutinous to feltrose, smoke-grey to pale brown with a light brown marginal area; aerial mycelium sparse, whitish to light brown, mainly in the central area; margins regular, moderately deep, reverse brown. Soluble pigment is yellowish to orange or absent; no exudates were observed (Figure 3). Colonies on MEAsw (Figure S2), reaching 22.5 mm in diameter after 28 days at 25 °C, are morphologically similar to PDAsw. Colonies on CMAsw (Figure S2) reaching 47.3 mm in diameter after 28 days at 25 °C, plane slightly umbonate, surface velutinous, olive-grey to pale brown with a large submerged peripheric area up to 10 mm, aerial mycelium, whitish to light brown, mainly in the central area, reverse brown. Soluble pigment and exudates not produced.

**Notes.** Based on a Megablast search on the NCBI nucleotide database, the closest hits of nrITS of *R. halocynthiae* (OR367549) are *R. gigliensis* (GenBank accession no. OR367423; identities 489/538 (91%), 20 gaps), *Lulworthia* sp. (GenBank accession no. KU214534; identities 464/532 (87%), 29 gaps), and *P. gigaspora* (GenBank accession no. MN649244; identities 467/536 (87%), 33 gaps). The closest hits using the nrLSU sequences are *R. gigliensis* (GenBank accession no. OR369725; identities 900/914 (98%), 4 gaps), *P. halima* (GenBank accession no. MT235754; identities 888/910 (98%), 0 gaps), and *P. posidoniae* (GenBank accession no. MZ357739; identities 887/909 (98%), 0 gaps). The closest hits using the nrSSU sequences are *L. uniseptata* (GenBank accession no. AY879034; identities 1039/1051 (99%), 0 gaps), *R. gigliensis* (GenBank accession no. OR371466; identities 1033/1043 (99%), 0 gaps), and *Z. maritima* (GenBank accession no. MT235710; identities 1038/1051 (99%), 0 gaps).

*R. halocynthiae* isolates can be collected from the tunic and the internal tissues of *H. papillosa* and can be cultured on media with and without sea salt; the best growth was observed at the sea salinity on Corn Meal Agar (CMAsw).

**Additional material examined**. Italy, Tuscany, Mediterranean Sea, Giglio Island (Grosseto), Punta Gabbianara, 42°21′50″ N, 10°55′24″ E, 25 m depth. Isolated from tunic or internal tissues of *H. papillosa*, March 2022, Marcella Pasqualetti, living culture HPa50, HPa54. Italy, Tuscany, Mediterranean Sea, Giglio Island (Grosseto), Punta Gabbianara, 42°21′50″ N, 10°55′24″ E, 28 m depth. Isolated from the tunic and internal tissues of *H. papillosa*, March 2022, Marcella Pasqualetti, living cultures HPa51, HPa53, HPa58, HPa59, HPa60, HPa61, HPa62, HPa63, and HPa64.

## 4. Discussion

Fungi are key players in terrestrial and marine environments and represent a substantial proportion of the microbial diversity on Earth [15]. Even if the role of marine fungi in several basic ecosystem functions, such as their contribution to aquatic carbon pump efficiency or regulation of phytoplankton composition, is largely recognized, the diversity of marine fungi seems to be largely unexplored. It was estimated that up to 90% of marine species have not been described yet [14]. Considering this gap, the exploration of habitats and substrates that have never been studied by mycologists appears to be an essential issue to enhance our knowledge of marine fungal biodiversity. Indeed, the new taxa proposed in this study were isolated from *H. papillosa*, a substratum that has never been previously studied from a mycological point of view.

The fifteen new isolates, obtained from the external tunic and internal tissues of the studied tunicate, developed only sterile mycelia. According to the literature, all strains were cultivated on different substrates, including artificial media (PDAsw, MEAsw, CMAsw, and OAsw), and natural matrices (bark, wood, and tunic of *H. papillosa*). To promote reproduction and the possible development of reproductive structures, the inoculated matrices were placed in both humid chambers and submerged in seawater during incubation [22,59,60,61]. Fungi development occurred in all studied conditions; nevertheless, sexual reproductive structures or asexual conidia have never been observed. Asexual chlamydospores were observed in 28-day-old cultures of *R. gigliensis* in all studied conditions, while *R. halocynthiae* produced vegetative mycelium only. *Mycelia sterilia* are not unusual among marine fungi [62,63], according to Damare and co-workers [64], it is possible that many marine fungi have evolved hyphal fragmentation as the preferential dispersion system. This would explain the broad presence of the toruloid mycelium observed in *R. gigliensis;* similar mycelia were reported for other Lulworthiales too [24].

Considering the absence of reproductive structures, except for the mentioned chlamydospores (propagules primarily devoted to perennation, not dissemination) in *R. gigliensis*, a molecular taxonomical approach was carried out for the taxonomical characterization of the identified strains. A preliminary analysis of the universal barcode for fungi (nrITS region) revealed similarity values inferior to 88% with all sequences deposited in the NCBI nucleotide database. This low identity clearly indicates that these strains were new taxa. Nevertheless, the ITS analyses indicated that all strains belonged to the order Lulworthiales, and the multi-locus molecular analyses, based on ribosomal genes (nrLSU, nrITS, and nrSSU), were performed to infer their phylogeny according to recent literature [21,22,23,24,25]. The phylogenetic tree clearly showed that our strains formed a well-supported clade that did not encompass any known fungus, indicating the presence of a new lineage inside the family Lulworthiaceae (Figure 1).

The order Lulworthiales includes only strictly marine species [20], commonly found in association with wood, seagrass, and algae. To the best of our knowledge, members of the order Lulworthiales have not been isolated from ascidians yet or from other marine animals [65,66,67,68]. The newly studied strains are epizoic, facultative halophytes. They can grow in media devoid of seawater, even if the optimal growth was observed at Mediterranean Sea salinity (38‰).

## 5. Conclusions

The present paper provides a morphological and phylogenetic study of fifteen strains obtained from the marine tunicate *Halocynthia papillosa* collected in the central Tyrrhenian Sea; this tunicate has never been studied for its mycobiota. The strains form a novel lineage within the family Lulworthiaceae. In light of this, the new genus *Rambellisea* has been established, including the two new species, *Rambellisea halocynthiae* sp. nov. and *Rambellisea gigliensis* sp. nov. The identification of fungi belonging to Lulworthiales significantly contributes to the advancement of knowledge about this order of marine species, confirming that the marine ecosystem constitutes an extensive repository of biodiversity, largely unexplored, in particular for its microbial components.

## Figures and Tables

**Table 1:** Details of PCR programs for the different markers used.

PCR Steps		nrITS	nrLSU	nrSSU
		ITS5/ITS4	LR0R/LR7	NS1/NS4
Initial denaturation		94 °C for 2′	95 °C for 10′	95 °C for 10′
PCR cycle	denaturation	94 °C for 20″	95 °C for 1′	95 °C for 1′
	annealing	56 °C for 30″	50 °C for 50″	50 °C for 50″
	elongation	72 °C for 45″	72 °C for 1.5′	72 °C for 1.5′
Final elongation		72 °C for 10′	72 °C for 10′	72 °C for 10′
Number of cycles		35	40	40

Legend: nrITS = nuclear ribosomal Internal Transcribed Spacer; nrLSU = nuclear ribosomal Large ribosomal SubUnit; nrSSU = nuclear ribosomal Small ribosomal SubUnit.

**Table 2:** Taxa used for the phylogenetic analyses and GenBank accession number. Newly generated sequences are indicated in bold.

Species	Strain	Substrates	nrITS	nrSSU	nrLSU
Lulworthiales					
*Cumulospora marina*	MF46	Submerged wood	-	GU252136	GU252135
	GC53	Submerged wood	-	GU256625	GU256626
*Cumulospora varia*	GR78	Submerged wood	-	EU848593	EU848578
	IT 152	Wood	-	EU848579	-
*Halazoon melhae*	MF819	Submerged wood	-	GU252144	GU252143
*Halazoon fuscus*	NBRC 105256	Driftwood	-	GU252148	GU252147
*Hydea pygmea*	NBRC 33069	Driftwood	-	GU252134	GU252133
	IT081	Driftwood	-	GU256632	GU256633
*Kohlmeyeriella crassa*	NBRC 32133	Driftwood	LC146741	-	LC146742
*Kohlmeyeriella tubulata*	PP1105	Sea foam	-	AY878998	AF491265
	PP0989	Marine environment	-	AY878997	AF491264
*Lindra marinera*	JK 5091A	Marine environment	-	AY879000	AY878958
*Lindra obtuse*	NRBC 31317	Sea foam	LC146744	AY879002	AY878960
	AFTOL 5012	Marine environment	-	FJ176847	FJ176902
	CBS 113030	-	-	AY879001	AY878959
*Lindra thalassiae*	JK 5090A	Marine environment	-	U46874	U46891
	AFTOL-ID 413	Marine environment	DQ491508	DQ470994	DQ470947
	JK 5090	Marine environment	-	AF195634	AF195635
	JK 4322	*Thalassia testudinum*	-	AF195632	AF195633
*Lulwoana uniseptate*	NBRC 32137	Submerged wood	LC146746	LC146746	LC146746
	CBS 16760	Driftwood	-	AY879034	AY878991
*Lulworthia atlantica*	FCUL210208SP4	Sea water	KT347205	KT347193	JN886843
	FCUL190407CF4	Sea water	KT347207	KT347198	JN886809
	FCUL061107CP3	Sea water	KT347208	KT347196	JN886825
*Lulworthia fucicola*	ATCC 64288	Intertidal wood	-	AY879007	AY878965
	PP1249	Marine environment	-	AY879008	AY878966
*Lulworthia fundyensis*	DAOMC 251940	Marine wood	NR_178138	-	-
	AW2347	Marine wood	MH465123	MH465136	MH458750
*Lulworthia grandispora*	AFTOL 424	Dead *Rhizophora* sp.	-	DQ522855	DQ522856
	NTOU3841	Driftwood	-	KY026044	KY026048
	NTOU3847	Mangrove wood	-	KY026046	KY026049
	NTOU3849	Mangrove wood	-	KY026047	KY026050
*Lulworthia medusa*	JK 5581	*Spartina* sp.	-	AF195636	AF195637
*Lulworthia opaca*	CBS 218.60	Driftwood	-	AY879003	AY878961
*Lulworthia* cf. *purpurea*	FCUL170907CP5	Seawater	KT347219	KT347201	JN886824
	FCUL280207CF9	Seawater	KT347218	KT347202	JN886808
*Matsusporium tropicale*	NBRC 32499	Submerged wood	-	GU252142	GU252141
*Paralulworthia candida*	MUT 5430	*P. oceanica*	MZ357724	MZ357767	MZ357746
*Paralulworthia elbensis*	MUT 377	*P. oceanica*	MZ357710	MZ357753	MZ357732
	MUT 5422	*P. oceanica*	MZ357723	MZ357766	MZ357745
	MUT 5438	*P. oceanica*	MZ357712	MZ357755	MZ357734
	MUT 5461	*P. oceanica*	MZ357725	MZ357768	MZ357747
*Paralulworthia gigaspora*	MUT 435	*P. oceanica*	MN649242	MN649246	MN649250
	MUT 5413	*P. oceanica*	MN649243	MN649247	MN649251
	MUT 263	Seawater	MZ357729	MZ357772	MZ357751
	MUT 465	*P. oceanica*	MZ357726	MZ357769	MZ357748
	MUT 1753	Seawater	MZ357730	MZ357773	MZ357752
	MUT 5085	*P. oceanica*	MZ357715	MZ357758	MZ357737
	MUT 5086	*P. oceanica*	MZ357716	MZ357759	MZ357738
	MUT 5093	*P. oceanica*	MZ357718	MZ357761	MZ357740
	MUT 5094	*P. oceanica*	MZ357719	MZ357762	MZ357741
*Paralulworthia halima*	CMG 68	Submerged wood	MT235736	MT235712	MT235753
	CMG 69	Submerged wood	MT235737	MT235713	MT235754
	MUT 1483	Submerged wood	MZ357727	MZ357770	MZ357749
	MUT 2919	Submerged wood	MZ357713	MZ357756	MZ357735
	MUT 3347	Submerged wood	MZ357728	MZ357771	MZ357750
*Paralulworthia mediterranea*	MUT 654	*P. oceanica*	MZ357711	MZ357754	MZ357733
	MUT 5080	*P. oceanica*	MZ357714	MZ357757	MZ357736
	MUT 5417	*P. oceanica*	MZ357721	MZ357764	MZ357743
*Paralulworthia posidoniae*	MUT 5261	*P. oceanica*	MN649245	MN649249	MN649253
	MUT 5092	*P. oceanica*	MZ357717	MZ357760	MZ357739
	MUT 5110	*P. oceanica*	MZ357720	MZ357763	MZ357742
	MUT 5419	*P. oceanica*	MZ357722	MZ357765	MZ357744
***Rambellisea halocynthiae***	**HPa50**	***H. papillosa***	**OR367481**	**OR371485**	**OR371457**
	**HPa51**	***H. papillosa***	**OR367548**	**OR371484**	**OR371461**
	**HPa52**	***H. papillosa***	**OR367549**	**-**	**OR371460**
	**HPa53**	***H. papillosa***	**OR367614**	**-**	**-**
	**HPa54**	***H. papillosa***	**OR378535**	**-**	**-**
	**HPa58**	***H. papillosa***	**OR367660**	**-**	**-**
	**HPa59**	***H. papillosa***	**OR367678**	**-**	**-**
	**HPa60**	***H. papillosa***	**OR367679**	**-**	**-**
	**HPa61**	***H. papillosa***	**OR367717**	**-**	**-**
	**HPa62**	***H. papillosa***	**OR378536**	**-**	**-**
	**HPa63**	***H. papillosa***	**OR378537**	**-**	**-**
	**HPa64**	***H. papillosa***	**OR378538**	**-**	**-**
***Rambellisea gigliensis***	**HPa3**	***H. papillosa***	**OR367423**	**OR371466**	**OR369726**
	**HPa15**	***H. papillosa***	**OR367447**	**OR371482**	**OR369725**
	**HPa16**	***H. papillosa***	**OR367450**	**OR371483**	**OR371456**
*Zalerion maritima*	FCUL280207CP1	Seawater	KT347216	KT347203	JN886806
	FCUL010407SP2	Seawater	KT347217	KT347204	JN886805
	CM66	Submerged wood	MT235734	MT235710	MT235751
	CM67	Submerged wood	MT235735	MT235711	MT235752
*Zalerion pseudomaritima*	CMG64	Submerged wood	MT235732	MT235708	MT235749
	CMG65	Submerged wood	MT235733	MT235709	MT235750
**Pleosporales**					
*Bimuria novae-zelandiae*	CBS107.79	soil	MH861181	AY016338	MH872950
*Setosphaeria monoceras*	CBS 154.26	-	DQ337380	DQ238603	AY016368
*Letendraea helminthicola*	CBS 884.85	Yerba mate	MK404145	AY016345	AY016362

## Data Availability

All data generated or analyzed during this study are included in this published article and its supplementary information files. All sequences were deposited in GenBank (https://www.ncbi.nlm.nih.gov/nuccore, accessed on 1 August 2023) and alignments were deposited at TreeBase (https://www.treebase.org/treebase-web/search/studySearch.html, accessed on 8 October 2023).

## Supplementary Materials

The following supporting information can be downloaded at https://www.mdpi.com/article/10.3390/jof10020127/s1, Table S1: Substitution rates, gamma distribution shape parameter and proportion of invariable sites for each partition, Table S2: The variable sites detected in the nrITS region among *Rambellisea halocynthiae*, *R. gigliensis* and its neighbor species belonging to the genera *Paralulworthia,* Table S3: The variable sites detected in the nrLSU region among *Rambellisea halocynthiae*, *R. gigliensis* and its neighbor species belonging to the genera *Paralulworthia,* Table S4: The variable sites detected in the nrSSU region among *Rambellisea halocynthiae*, *R. gigliensis* and its neighbor species belonging to the genera *Paralulworthia,* Figure S1: *Rambellisea gigliensis* sp. nov. HPa3 (MUT 6843). (a) 28-day-old colony: colony texture on MEAws at 25 °C; (b) 28-day-old colony: colony texture on CMAws at 25 °C, Figure S2: *Rambellisea halocynthiae* sp. nov. HPa52 (MUT 6851). (a) 28-day-old colony on MEAws (Ø 9 cm) at 25 °C (b) and reverse; (c) 28-day-old colony on CMAws (Ø 9 cm) at 25 °C (d) and reverse.
