# Supplementary material for: Rambellisea gigliensis and Rambellisea halocynthiae, gen. et spp. nov. (Lulworthiaceae) from the Marine Tunicate Halocynthia papillosa
Source: J Fungi (Basel). 2024 Feb 3;10(2):127. doi: 10.3390/jof10020127 (PMC10890369; doi:10.3390/jof10020127)
Supplement: Supplementary file 1 [file jof-10-00127-s001.zip › jof-2756954-supplementary.pdf]

Journal of *Fungi*

Supplementary Materials

Rambellisea gigliensis and *Rambellisea halocynthiae*, gen. et spp. nov. (Lulworthiaceae) from the Marine Tunicate *Halocynthia papillosa*

Martina Braconcini [1], Susanna Gorrasi [1], Massimiliano Fenice [1,2], Paolo Barghini [1] and Marcella Pasqualetti [1,3,*]

[1] Department of Ecological and Biological Sciences (DEB), University of Tuscia, 01100 Viterbo, Italy; martina.braconcini@unitus.it (M.B.); gorrasi@unitus.it (S.G.); fenice@unitus.it (M.F.); barghini@unitus.it (P.B.)
[2] Laboratory of Applied Marine Microbiology, CoNISMa, Department of Ecological and Biological Sciences, University of Tuscia, 01100 Viterbo, Italy
[3] Laboratory of Ecology of Marine Fungi, CoNISMa, Department of Ecological and Biological Sciences, University of Tuscia, 01100 Viterbo, Italy
* Correspondence: mpasqual@unitus.it; Tel.: +39-0761-357750

List of Contents

Table S1. Substitution rates, gamma distribution shape parameter and proportion of invariable sites for each partition

Table S2. The variable sites detected in the nrITS region among *Rambellisea halocynthiae*, *R. gigliensis* and its neighbor species belonging to the genera *Paralulworthia.*

Table S3. The variable sites detected in the nrLSU region among *Rambellisea halocynthiae*, *R. gigliensis* and its neighbor species belonging to the genera *Paralulworthia.*

Table S4. The variable sites detected in the nrSSU region among *Rambellisea halocynthiae*, *R. gigliensis* and its neighbor species belonging to the genera *Paralulworthia.*

Figure S1. *Rambellisea gigliensis* sp. nov. HPa3 (MUT 6843). (a) 28-day-old colony: colony texture on MEAws at 25° C; (b) 28-day-old colony: colony texture on CMAws at 25° C.

Figure S2. *Rambellisea halocynthiae* sp. nov. HPa52 (MUT 6851). (a) 28-day-old colony on MEAws (Ø 9 cm) at 25° C (b) and reverse; (c) 28-day-old colony on CMAws (Ø 9 cm) at 25° C (d) and reverse

Citation: Braconcini, M.; Gorrasi, S.; Fenice, M.; Barghini, P.; Pasqualetti, M. *Rambellisea gigliensis* and *Rambellisea halocynthiae*, gen. et spp. nov. (Lulworthiaceae) from the Marine Tunicate *Halocynthia papillosa*. *J. Fungi* **2024**, *10*, 127. https://doi.org/10.3390/jof10020127

Academic Editor: Philippe Silar

Received: 20 November 2023
Revised: 24 January 2024
Accepted: 1 February 2024
Published: 3 February 2024

Copyright: © 2024 by the authors. Licensee MDPI, Basel, Switzerland. This article is an open access article distributed under the terms and conditions of the Creative Commons Attribution (CC BY) license (https://creativecommons.org/licenses/by/4.0/).

Table S1. Substitution rates, gamma distribution shape parameter and proportion of invariable sites for each partition

	Substitution rates	Gamma distribution shape	Proportion of invariable site
**Partition 1**	gamma	0.6400	0.3970
**Partition 2**	gamma	0.3760	0
**Partition 3**	gamma	1.7790	0
**Partition 4**	gamma	0.2890	0
**Partition 5**	gamma	1.1200	0
**Partition 6**	gamma	0.3890	0

Table S2. The variable sites detected in the nrITS region among *Rambellisea halocynthiae*, *R. gigliensis* and its neighbor species belonging to the genera *Paralulworthia.*

**Species**	**Position**																
	**20**	**23**	**24**	**44**	**46**	**54**	**56**	**57**	**94**	**101**	**102**	**103**	**104**	**108**	**109**	**113**	**114**
R. halocynthiae	C	-	-	T	G	T	G	G	C	C	T	C	C	A	-	T	C
R. gigliensis	C	-	-	C	G	A	A	A	C	A	A	A	A	A	C	T	C
P. candida	-	A	A	T	A	-	T	G	A	C	A	C	G	C	A	C	A
P. elbensis	-	A	A	T	A	T	T	G	A	C	A	C	G	C	A	C	A
P gigaspora	A	A	A	T	A	T	T	G	A	C	A	C	C	C	A	C	A
P. halima	A	A	A	T	A	T	T	G	A	C	A	C	C	C	A	C	A
P. mediterranea	A	A	A	T	A	T	T	G	A	C	A	C	C	C	A	C	A
P. posidoniae	A	A	A	T	A	T	T	G	A	C	A	C	C	C	A	C	A

**Species**	**Position**																
	**119**	**120**	**121**	**122**	**123**	**129**	**131**	**132**	**159**	**170**	**171**	**245**	**317**	**329**	**340**	**365**	**366**
R. halocynthiae	C	T	A	C	G	T	A	A	A	A	A	A	A	T	T	C	T
R. gigliensis	C	T	G	C	A	C	A	A	A	A	G	A	A	T	C	T	T
P. candida	A	C	C	A	G	T	C	C	G	C	A	C	G	C	T	-	-
P. elbensis	A	C	C	A	G	T	C	C	G	C	A	C	G	C	T	-	-
P gigaspora	A	C	C	A	G	T	C	C	G	C	A	C	G	C	T	C	C
P. halima	A	C	C	A	G	T	C	C	G	C	A	C	G	C	T	C	C
P. mediterranea	A	C	C	A	G	T	C	C	G	C	A	C	G	C	T	C	C
P. posidoniae	A	C	C	A	G	T	C	C	G	C	A	C	G	C	T	C	C

**Species**	**Position**																
	**368**	**369**	**370**	**371**	**373**	**374**	**376**	**377**	**384**	**385**	**386**	**400**	**429**	**430**	**439**	**447**	**464**
R. halocynthiae	T	C	C	T	C	G	G	A	T	G	G	C	A	A	T	C	C
R. gigliensis	C	T	T	C	C	A	G	A	C	G	T	T	C	A	G	C	C
P. candida	A	C	C	T	T	T	C	G	T	A	G	C	T	C	G	T	T
P. elbensis	A	C	C	T	T	T	C	G	T	A	G	C	T	C	G	T	T
P gigaspora	A	C	C	T	T	T	C	G	T	A	G	C	T	C	G	T	T
P. halima	A	C	C	T	T	T	C	G	T	A	G	C	T	C	G	T	T
P. mediterranea	A	C	C	T	T	T	C	G	T	A	G	C	T	C	G	T	T
P. posidoniae	A	C	C	T	T	T	C	G	T	A	G	C	T	C	G	T	T

**Species**	**Position**																
	**465**	**470**	**474**	**486**	**487**	**488**	**509**	**512**	**513**	**514**	**515**	**516**	**531**	**532**	**576**	**578**	**582**
R. halocynthiae	C	C	T	-	-	-	G	T	C	G	G	T	G	A	T	C	C
R. gigliensis	T	G	T	-	-	-	G	T	C	G	G	T	G	A	C	C	G
P. candida	C	T	C	C	C	A	A	C	A	-	-	-	A	G	C	A	C
P. elbensis	C	T	C	C	C	A	A	C	A	-	-	-	A	G	C	A	C

P gigaspora	C	C	C	C	C	A	A	C	A	-	-	-	A	G	C	A	C
P. halima	C	C	C	C	C	A	A	C	A	-	-	-	A	G	C	A	C
P. mediterranea	C	C	C	C	C	A	A	C	A	-	-	-	A	G	C	A	C
P. posidoniae	C	C	C	C	C	A	A	C	A	-	-	-	A	G	C	A	C

	588	589	600
R. halocynthiae	A	C	**A**
R. gigliensis	**C**	**T**	**A**
P. candida	A	C	C
P. elbensis	A	C	C
P gigaspora	A	C	C
P. halima	A	C	C
P. mediterranea	A	C	C
P. posidoniae	A	C	C

Table S3. The variable sites detected in the nrLSU region among *Rambellisea halocynthiae*, *R. gigliensis* and its neighbor species belonging to the genera *Paralulworthia*..

**Species**	**Position**																
	**102**	**144**	**145**	**173**	**383**	**407**	**408**	**466**	**467**	**468**	**469**	**487**	**488**	**508**	**509**	**540**	**569**
R. halocynthiae	**T**	**G**	G	C	C	**T**	**C**	**C**	**G**	**A**	**T**	**T**	**T**	**C**	**T**	T	**T**
R. gigliensis	**T**	**A**	**C**	**T**	**A**	**T**	**C**	T	**G**	**A**	C	C	C	**C**	**T**	**C**	C
P. candida	C	T	G	C	C	-	-	T	A	G	C	C	C	T	C	T	C
P. elbensis	C	T	G	C	C	-	-	T	A	G	C	C	C	T	C	T	C
P gigaspora	C	T	G	C	C	-	-	T	A	G	C	C	C	T	C	T	C
P. halima	C	T	G	C	C	-	-	T	A	G	C	C	C	T	C	T	C
P. mediterranea	C	T	G	C	C	-	-	T	A	G	C	C	C	T	C	T	C
P. posidoniae	C	T	G	C	C	-	-	T	A	G	C	C	C	T	C	T	C

**Species**	**Position**															
	**601**	**643**	**668**	**764**	**765**	**772**	**808**	**809**	**878**	**1016**	**1050**	**1051**	**1052**	**1062**	**1070**	**1071**
R. halocynthiae	**T**	**T**	**A**	**A**	**A**	**A**	**T**	**A**	**T**	**T**	C	**C**	**T**	**G**	T	**G**
R. gigliensis	**T**	**T**	**A**	**A**	**A**	**A**	**T**	**A**	**T**	**T**	**T**	**C**	**T**	**G**	**C**	**G**
P. candida	C	C	C	G	G	G	C	G	C	C	C	A	C	A	T	C
P. elbensis	C	C	C	G	G	G	C	G	C	C	C	A	C	A	T	C
P gigaspora	C	C	C	G	G	G	C	G	C	C	C	A	C	A	T	C
P. halima	C	C	C	G	G	G	C	G	C	C	C	A	C	A	T	C
P. mediterranea	C	C	C	G	G	G	C	G	C	C	C	A	C	A	T	C
P. posidoniae	C	C	C	G	G	G	C	G	C	C	C	A	C	A	T	C

Table S4. The variable sites detected in the nrSSU region among *Rambellisea halocynthiae*, *R. gigliensis* and its neighbor species belonging to the genera *Paralulworthia.*

**Species**	**Position**																
	**43**	**99**	**100**	**101**	**107**	**110**	**121**	**129**	**130**	**140**	**161**	**219**	**323**	**349**	**350**	**376**	**1126**
R. halocynthiae	**T**	T	C	G	**A**	**G**	**A**	C	**T**	**T**	**T**	**A**	**A**	**A**	**G**	**C**	**T**
R. gigliensis	**T**	**A**	**A**	**C**	**C**	T	G	**T**	**T**	**T**	**T**	**A**	**A**	G	A	T	**T**

P. candida	G	T	C	G	G	T	G	C	A	C	C	G	T	G	A	T	A
P. elbensis	G	T	C	G	G	T	G	C	A	C	C	G	T	G	A	T	A
P gigaspora	G	T	C	G	G	T	G	C	A	C	C	G	T	G	A	T	A
P. halima	G	T	C	G	G	T	G	C	A	C	C	G	T	G	A	T	A
P. mediterranea	G	T	C	G	G	T	G	C	A	C	C	G	T	G	A	T	T
P. posidoniae	G	T	C	G	G	T	G	C	A	C	C	G	T	G	A	T	A

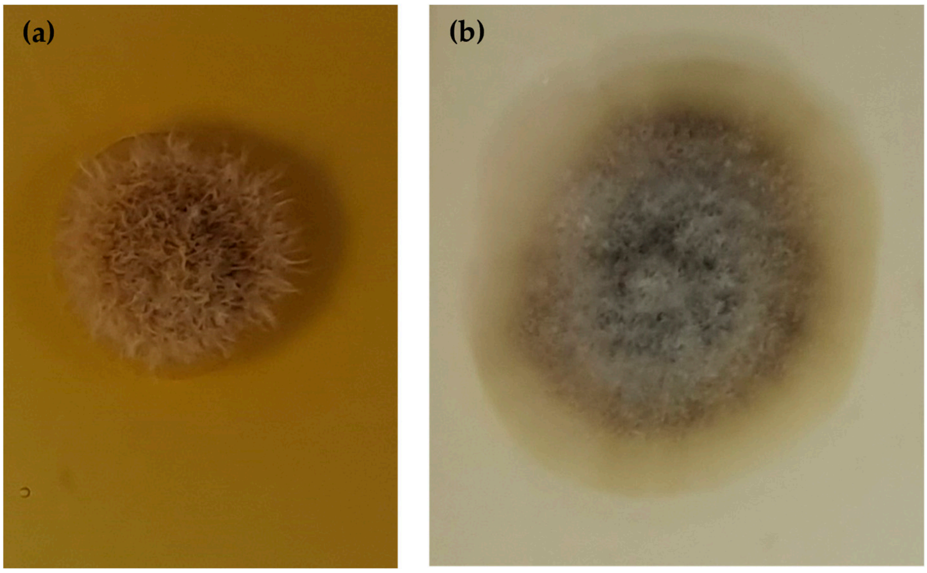

Figure S1. *Rambellisea gigliensis* sp. nov. HPa3 (MUT 6843). (a) 28-day-old colony: colony texture on MEAws at 25° C; (b) 28-day-old colony: colony texture on CMAws at 25° C.

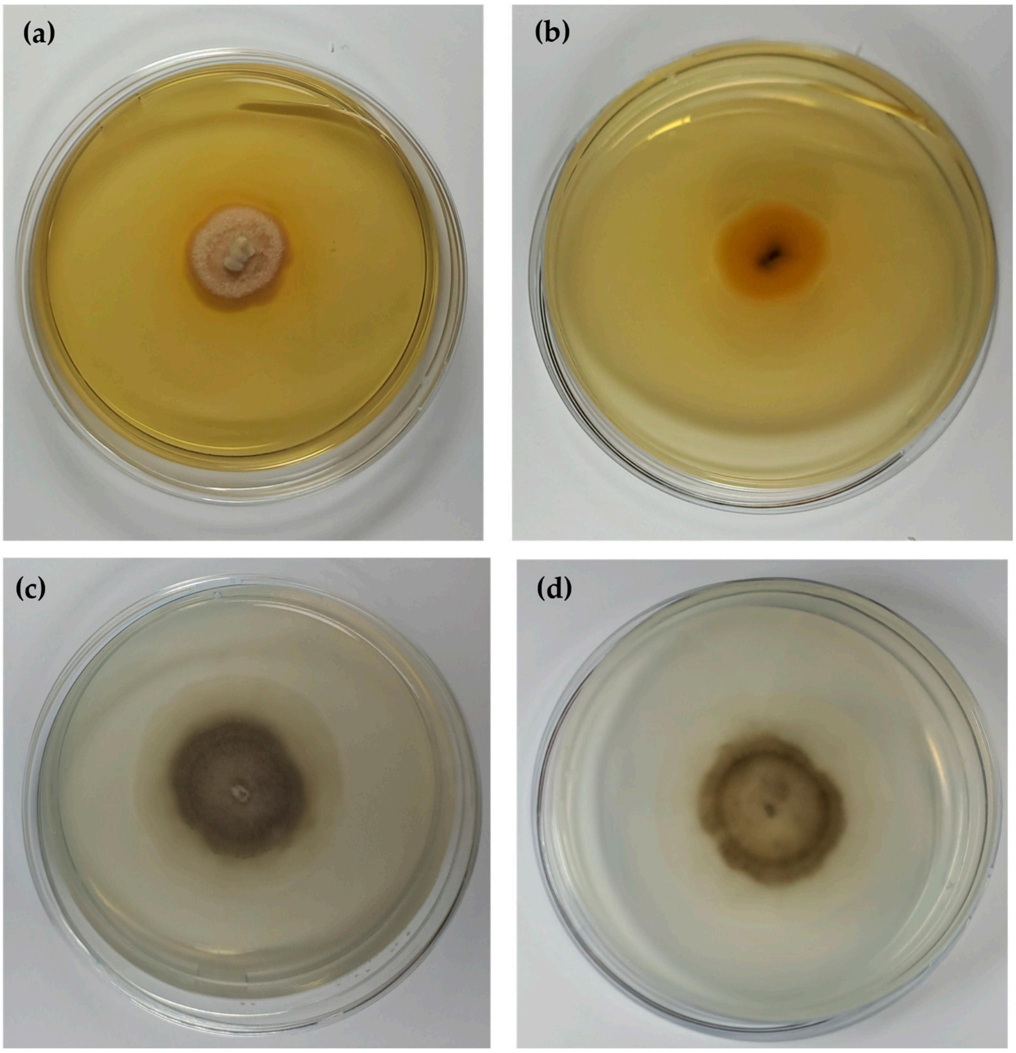

Figure S2. *Rambellisea halocynthiae* sp. nov. HPa52 (MUT 6851). (a) 28-day-old colony on MEAws (Ø 9 cm) at 25° C (b) and reverse; (c) 28-day-old colony on CMAws (Ø 9 cm) at 25° C (d) and reverse.
